# A laminar flow model of aerosol survival of epidemic and non-epidemic strains of *Pseudomonas aeruginosa *isolated from people with cystic fibrosis

**DOI:** 10.1186/1471-2180-8-105

**Published:** 2008-06-26

**Authors:** Ian J Clifton, Louise A Fletcher, Clive B Beggs, Miles Denton, Daniel G Peckham

**Affiliations:** 1 Regional Cystic Fibrosis Unit, St James University Hospital, Leeds, UK; 2 Department of Civil Engineering, University of Leeds, Leeds, UK; 3 Bradford Infection Group, School of Engineering, Design and Technology, University of Bradford, Bradford, BD7 1DP, UK; 4 Department of Microbiology, Leeds General Infirmary, Great George Street, Leeds, UK

## Abstract

**Background:**

Cystic fibrosis (CF) is an inherited multi-system disorder characterised by chronic airway infection with pathogens such as *Pseudomonas aeruginosa*.

Acquisition of *P. aeruginosa *by patients with CF is usually from the environment, but recent studies have demonstrated patient to patient transmission of certain epidemic strains, possibly via an airborne route. This study was designed to examine the survival of *P. aeruginosa *within artificially generated aerosols.

**Results:**

Survival was effected by the solution used for aerosol generation. Within the aerosols it was adversely affected by an increase in air temperature. Both epidemic and non-epidemic strains of *P. aeruginosa *were able to survive within the aerosols, but strains expressing a mucoid phenotype had a survival advantage.

**Conclusion:**

This would suggest that segregating individuals free of *P. aeruginosa *from those with chronic *P. aeruginosa *infection who are more likely to be infected with mucoid strains may help reduce the risk of cross-infection. Environmental factors also appear to influence bacterial survival. Warming and drying the air within clinical areas and avoidance of humidification devices may also be beneficial in reducing the risk of cross-infection.

## Background

Cystic fibrosis (CF) is an inherited multi-system disorder characterised by chronic airway infection, pancreatic insufficiency, elevated sweat chloride concentration, impaired fertility and hepatobiliary disease. The condition is due to mutations in the cystic fibrosis transmembrane conductance regulator (CFTR). Lack of CFTR function results in reduced fluid secretion and excessive fluid absorption, with a net effect of producing a thickening of the mucous component of the airway surface liquid. This causes plugging of the sub-mucosal glands and impairment of mucociliary clearance leading to infection, inflammation, and tissue damage resulting in bronchiectasis and a predisposition to infection with pathogens such as *Pseudomonas aeruginosa*.

*P. aeruginosa *is the most common and clinically important pathogen in patients with CF. The organism is a Gram-negative, non-fermentative, aerobic bacillus belonging to the family Pseudomonadaceae. Although classified as an aerobic organism *P. aeruginosa *is a facultative anaerobe which may allow it to survive within the relatively hypoxic lungs of patients with CF. It is ubiquitous within the environment and is particularly isolated from moist areas such as soil and water. The bacterium is frequently found in environmental reservoirs, such as the drains of hospital ward wash basins [1] and aerosols containing *P. aeruginosa *can be detected when opening taps [2,3]. The isolation of *P. aeruginosa *from tap water follows contamination of the taps rather than the mains water supply [4].

Acquisition of *P. aeruginosa *infection in patients with CF can occur at any age. During early stages of *P. aeruginosa *infection in patients with CF the bacteria are not usually mucoid and can be cleared with aggressive antibiotic treatment [5]. Once the patients lung becomes chronically infected the bacteria change to a mucoid phenotype and form biofilms. Most studies would suggest that 70–80% of patients are infected during the teenage years [6]. A number of studies have also demonstrated an association between chronic *P. aeruginosa *infection and increased mortality [7-9]. Acquisition of *P. aeruginosa *infection by patients with CF is usually from the environment, but recent studies have demonstrated patient to patient transmission of certain strains of *P. aeruginosa *[10-13]. More worryingly, some of these epidemic strains are associated with increased morbidity and treatment burden when compared to non-epidemic strains [14,15]. While the precise method of transmission of epidemic strains of *P. aeruginosa *remains unclear, there is evidence that airborne transmission may be important [16,17] and therefore a study was designed to investigate this further. The aims of the study were to: 1) examine the effects of varying environmental conditions on the survival of *P. aeruginosa *within artificially generated aerosols; and 2) identify differences in airborne survival of epidemic and non-epidemic strains under controlled environmental conditions.

## Methods

### Bacterial strains

*P. aeruginosa *(NCIMB 10848) was obtained from the National Collection of Industrial and Marine Bacteria. Isolates of both epidemic and non-epidemic strains of *P. aeruginosa *were obtained from stored strains at the Department of Microbiology, Leeds General Infirmary and the Centre for Infectious Diseases, University of Edinburgh (See Table 1). The clinical strains of *P. aeruginosa *used in this study were stored isolates originally collected from patients with cystic fibrosis and genotyped as part of routine clinical care and cross-infection surveillance. During this study no samples of sputum were obtained from human subjects, therefore ethical approval was not deemed to be necessary.

**Table 1:** Bacterial strains.

Bacterial strain	Mucoid phenotype	Epidemic strain	Strain identification
Environmental	No	No	NCIMB 10848
Unique CF	No	No	4412061*
Unique CF mucoid	Yes	No	43903641*
Manchester	No	Yes	Jones et al [10]‡
Leeds Seacroft 1	No	Yes	4390416-2*
Leeds Seacroft 2	No	Yes	4390195*
Leeds Paediatric	No	Yes	Denton et al [12]*
Leeds Paediatric mucoid	Yes	Yes	Denton et al [12]*
Liverpool	No	Yes	McCallum et al [11]‡
Liverpool mucoid	Yes	Yes	McCallum et al [11]‡

*Stored strains obtained from the Department of Microbiology, Leeds General Infirmary originally isolated from people with cystic fibrosis identified through routine infection control surveillance to be epidemic or non-epidemic; ‡Strains obtained from Centre for Infectious Diseases, University of Edinburgh

### Preparation of frozen bacterial cultures

Bacteria of each strain examined were harvested from 200 ml of overnight, stationary liquid broth culture, washed, and stored in 5 × 1 mL Eppendorf tubes with 40% (v v^-1^) glycerol solution. The concentrated bacterial suspensions were stored at -20°C until required.

### Aerosol generation

All aerosol generation was undertaken in a negatively pressurised Class II aerobiological chamber. Aerosols were generated from 100 mL nebuliser solutions containing 10^5 ^CFU mL^-1 ^of bacteria prepared from the frozen bacterial cultures, using a Collison 3-jet nebuliser [18] (BGI, USA) operating at 6 L min^-1 ^and 103.4 kPa. The concentration of bacteria within the nebuliser suspension was determined both pre- and post-nebulisation using serial dilution.

### Laminar flow model

**Figure 1:**
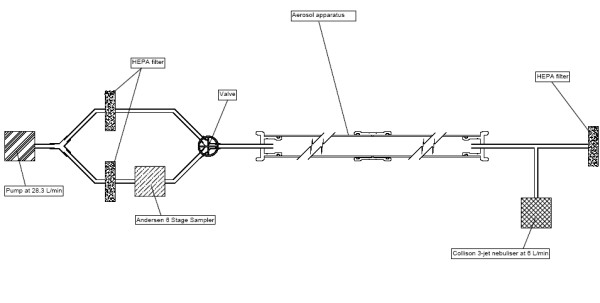
Laminar flow model

The aerosols were delivered into a 110 mm diameter air-tight pipe with a variable length (See Figure 1). The relative humidity and temperature of the air within the apparatus were controlled. In order to ensure a steady state condition, samples were taken only after the apparatus had run for 10 min. Thereafter, air samples were taken at 5 min intervals. Unless otherwise specified, relative humidity and temperature were maintained at 45% ± 2% and 22°C ± 2°C. All experiments were undertaken in triplicate. The velocity of the air within the laminar flow apparatus was calculated to be 0.0491 m sec^-1^.

### Air sampling

During each sampling event 56.6 L of air was drawn (i.e. at a rate of 28.3 L min^-1^) through an Andersen 6-stage impactor [19] (Andersen Inc, USA) containing nutrient agar plates which were then incubated at 37°C for 24 hr. The numbers of colonies on each plate were counted, and the count corrected using published positive-hole correction tables [20]. The concentration of viable bacteria in the air sample was then calculated. During experimentation the length of the laminar flow apparatus was varied and air samples were taken in triplicate at lengths of 2, 3 and 4 m, which equates to mean aerosol ages of 40.3, 60.4 and 80.6 sec, respectively. In order to determine the size distribution of the droplet nuclei generated, Stages 1–6 of the Andersen sampler were used when the length of the laminar flow apparatus length was 2 m. Thereafter, stages 5 and 6 only were used for apparatus lengths of 3 m and 4 m.

### Experimental variables

In order to assess the impact of variations in the nebuliser suspension fluid, aerosols were generated into the laminar flow apparatus using variously 100 mL of distilled water, 1/8 × Ringers, 1/4 × Ringers, 1/2 × Ringers, 1 × Ringers and 2 × Ringers solutions, and 10% (v v^-1^) foetal bovine serum (FBS) containing 10^5 ^CFU mL^-1 ^of *P. aeruginosa *(NCIMB 10848) inoculated from the same frozen sample.

In order to assess the impact of variations in temperature on microbial survival, the temperature of the air within the laminar flow model was maintained at either 22 ± 2°C or 27 ± 2°C, and 45% Relative Humidity (RH) by adjustment of the air conditioning controls of the class II aerobiological chamber. Aerosols were generated into the laminar flow model using 100 mL of 1/4 × Ringers containing 10^5 ^CFU mL^-1 ^of *P. aeruginosa *(NCIMB 10848).

In order to assess the impact of variations in air humidity on microbial survival, the relative humidity of the air within the laminar flow model was maintained at either 45% RH or 67% RH, and 22 ± 2°C by adjustment of the air conditioning controls of the class II aerobiological chamber. Aerosols were generated into the laminar flow model using 100 mL of 1/4 × Ringers containing 10^5 ^CFU mL^-1 ^of *P. aeruginosa *(NCIMB 10848).

In order to examine the survival of strains of *P. aeruginosa *in the laminar flow model aerosols were generated into the laminar flow model using 100 mL of 1/4 × Ringers containing 10^5 ^CFU mL^-1 ^of each strain of *P. aeruginosa*.

### Statistical analysis

All data was expressed as a mean and standard error of mean (SEM) The t-Test and one-way ANOVA was used for analysis of different groups (GraphPad 5.01, GraphPad Software Inc.). A p-value of < 0.05 was taken to be significant.

## Results

### Effect of nebuliser solution on survival of P. aeruginosa in aerosols

**Figure 2:**
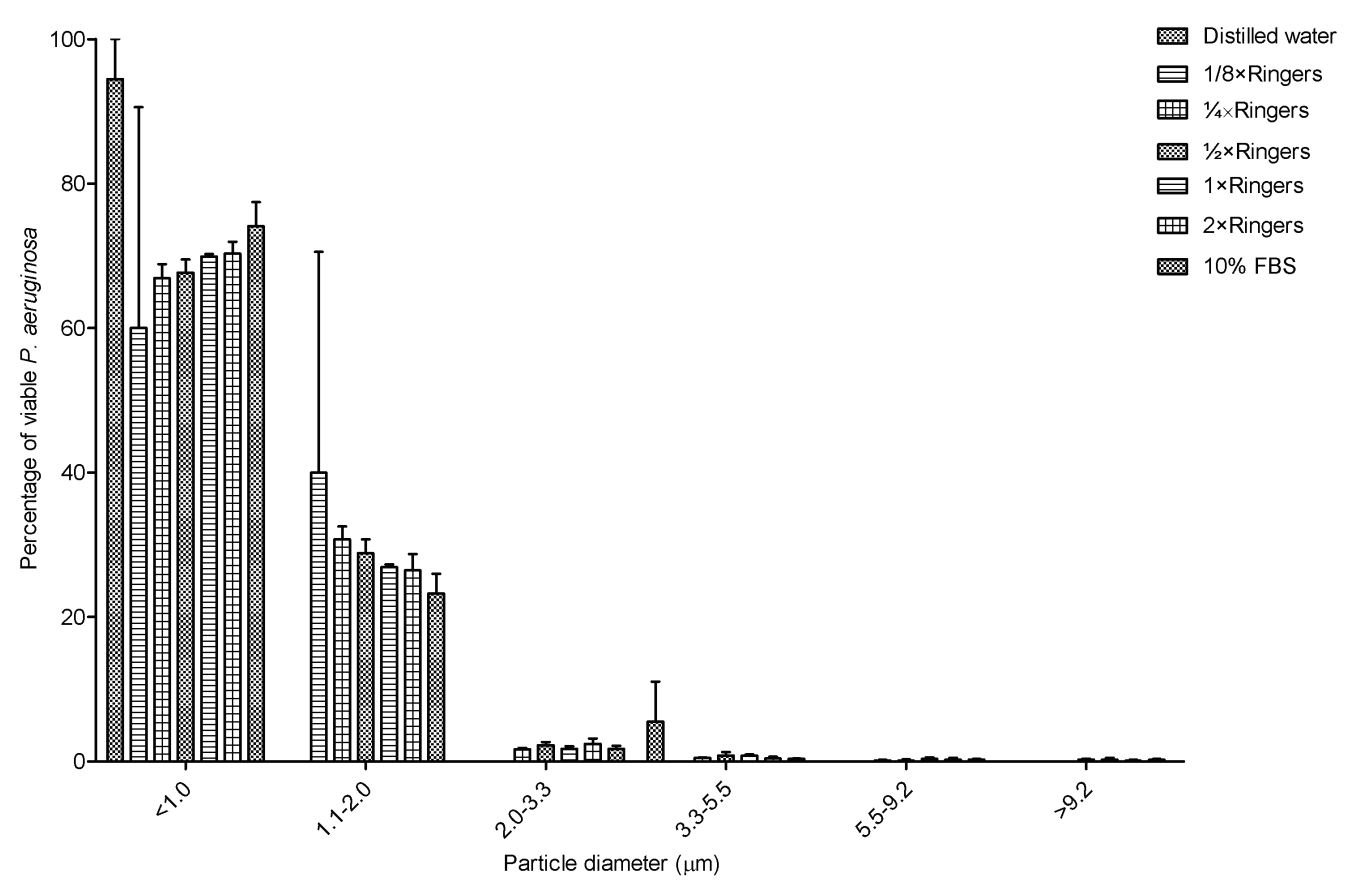
**Size distribution of particles sampled from aerosols containing *P. aeruginosa *(NCIMB 10848) generated using distilled water, different Ringer's solutions and 10% FBS.** Error bars represent standard error of mean.

Aerosols containing *P. aeruginosa *(NCIMB 10848) when generated using distilled water, 1/8 × Ringers, 1/4 × Ringers, 1/2 × Ringers, 1 × Ringers and 2 × Ringers solutions, and 10% FBS predominantly produce aerosols containing particles of less than 2.0 μm in diameter (See Figure 2). There was no significant difference between the percentage of particles with a diameter less than 2.0 μm between the aerosols generated using the different nebuliser solutions (One-way ANOVA p = 0.7404).

**Figure 3:**
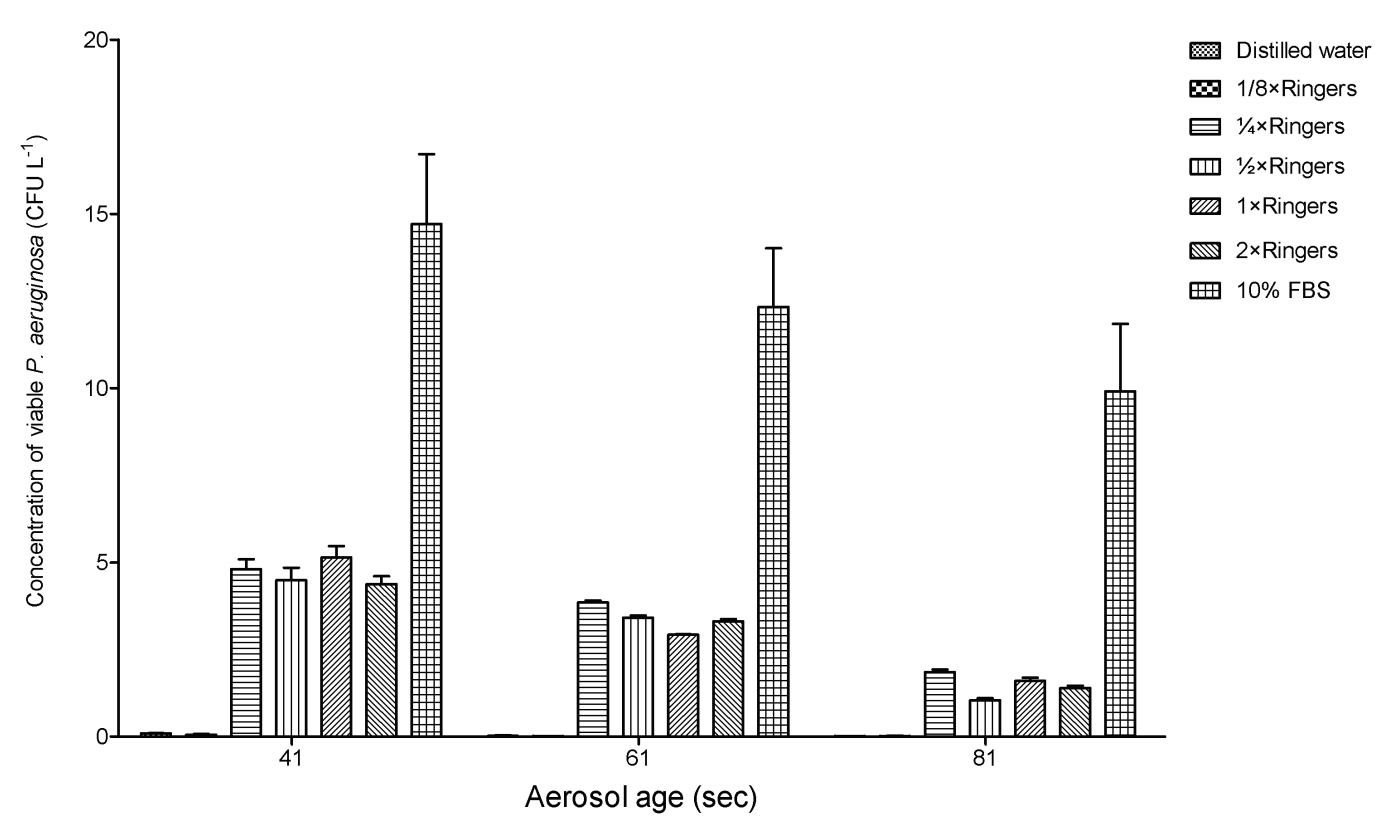
**Concentration of viable *P. aeruginosa *(NCIMB 10848) at different aerosol ages generated using distilled water, different Ringer's solutions and 10% FBS.** Error bars represent standard error of mean.

There was no significant difference between the concentration of viable *P. aeruginosa *(NCIMB 10848) in the aerosols generated using 1/4 × Ringers and 1/2 × Ringers (p = 0.0705), or 1 × Ringers (p = 0.5198), or 2 × Ringers (p = 0.055). Generation of aerosols using hypotonic solutions resulted in a significant reduction in the concentration of viable *P. aeruginosa *(NCIMB 10848) isolated from the aerosols compared to the use of isotonic Ringer's solution (Distilled water v 1/4 × Ringers = 0.0276; 1/8 × Ringers v 1/4 × Ringers p = 0.0282). The concentration of viable *P. aeruginosa *(NCIMB 10848) was significantly increased when the aerosols generated using 10% FBS compared to 1/4 × Ringers (p = 0.0040) (See Figure 3).

### Effect of environmental conditions on survival of P. aeruginosa in aerosols

**Figure 4:**
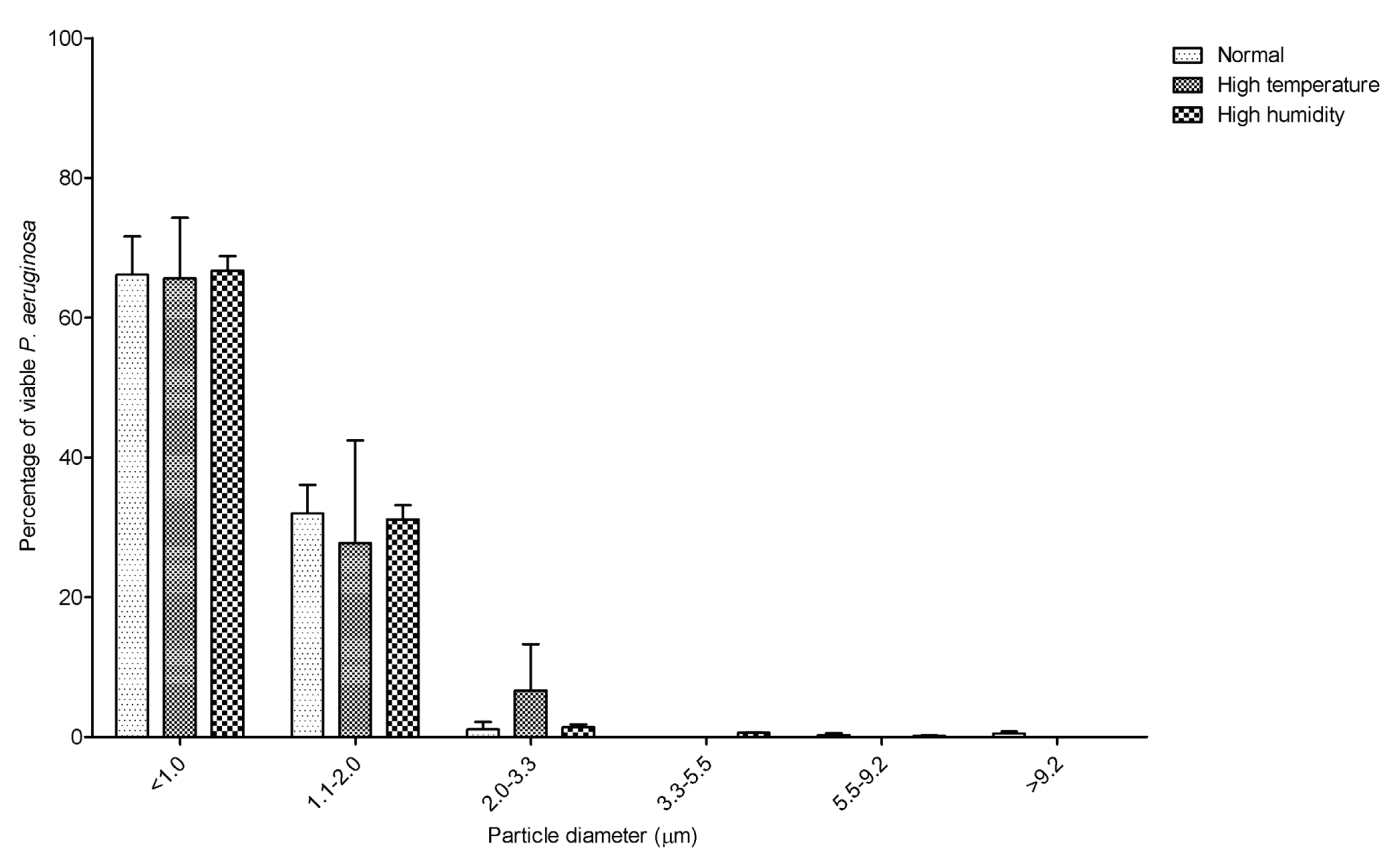
**Size distribution of particles sampled from aerosols containing *P. aeruginosa *(NCIMB 10848) generated using Ringer's solutions into conditions of normal temperature and humidity (22°C 45% RH), high temperature (27°C 45% RH) or high humidity (22°C 67%RH).** Error bars represent standard error of mean.

Conditions of high temperature or humidity did not affect the size distribution of the aerosols. There was no change in the size distribution of aerosol particles generated under standard conditions (22°C, 45%RH), compared with conditions of high temperature (27°C, 45% RH) and high humidity (22°C. 67%RH). Under all three environmental conditions the aerosols generated predominately contained particles less than 2.0 μm diameter (See Figure 4). There was no significant difference between the three environmental conditions in terms of percentage of particles less than 2.0 μm (One-way ANOVA p = 0.6508).

**Figure 5:**
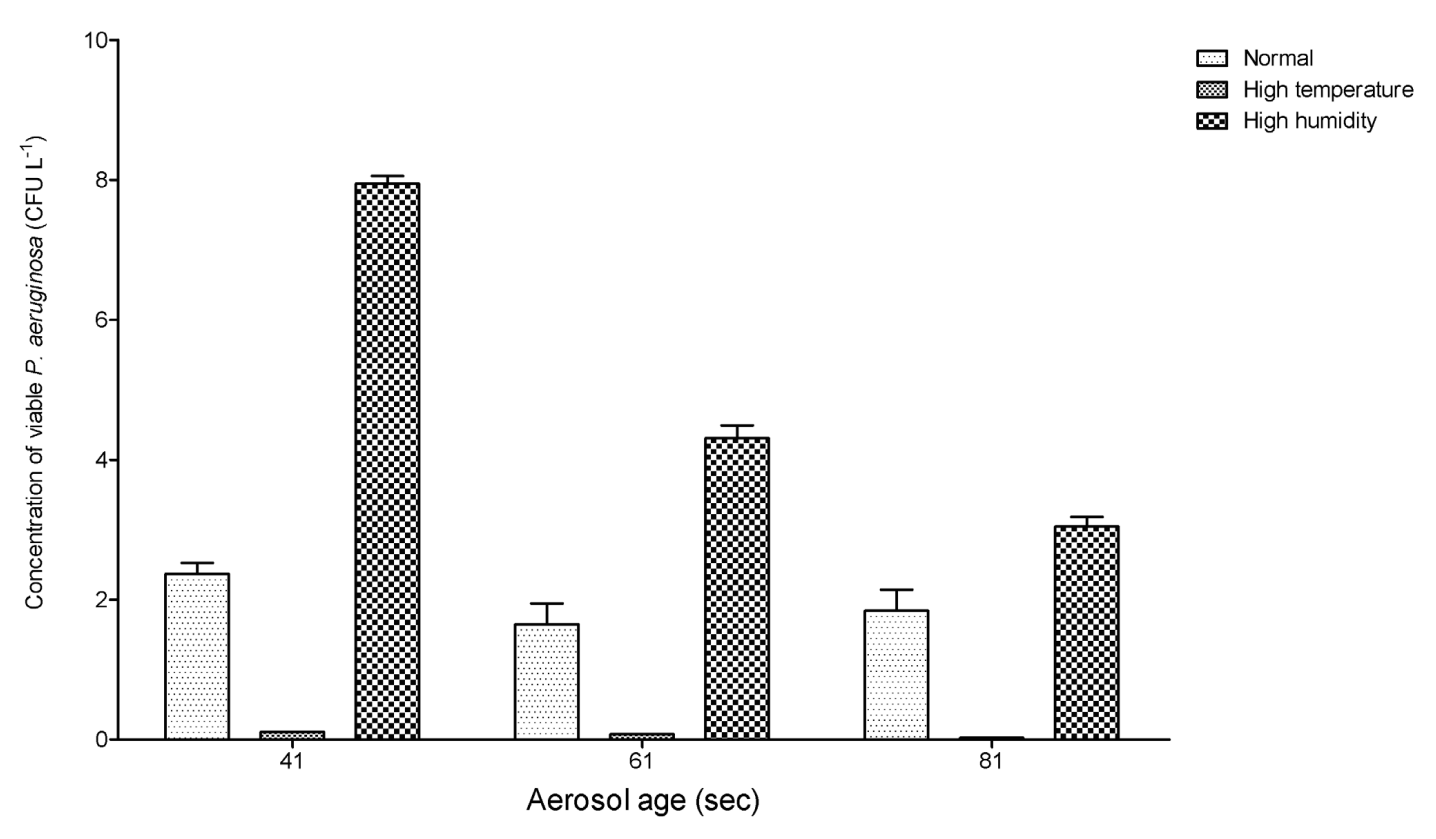
**Concentration of viable *P. aeruginosa *(NCIMB 10848) at different aerosol ages generated using Ringer's solutions into conditions normal temperature and humidity (22°C 45% RH), high temperature (27°C 45% RH) or high humidity (22°C 67%RH).** Error bars represent standard error of mean.

Generation of aerosols under conditions of increased temperature resulted in a significant decrease in the concentration of viable *P. aeruginosa *(p = 0.0114). The concentration of viable *P. aeruginosa *was greater under conditions of high humidity, but this did not reach statistical significance (p = 0.1340) (See Figure 5).

### Effect of different strains of P. aeruginosa in aerosols

**Figure 6:**
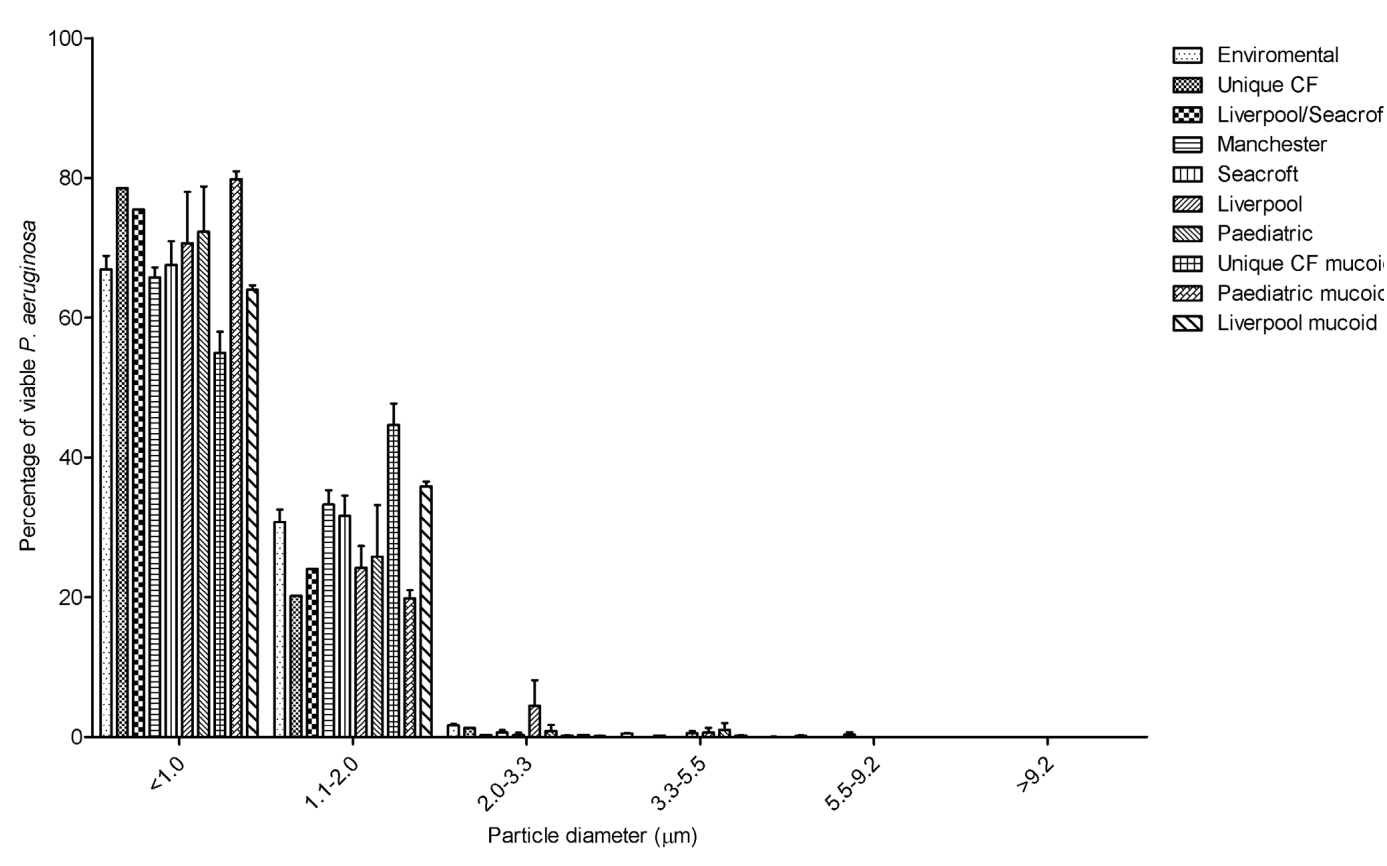
**Size distribution of particles sampled from aerosols containing different strains of *P. aeruginosa *generated using Ringer's solutions.** Error bars represent standard error of mean.

All the strains of *P. aeruginosa *had similar aerosol particle size distributions. All 10 strains tested produced aerosols predominately containing particles less than 2.0 μm diameter (See Figure 6). There was no significant difference between the 10 strains in terms of percentage of particles less than 2.0 μm (One-way ANOVA p = 0.4201).

**Figure 7:**
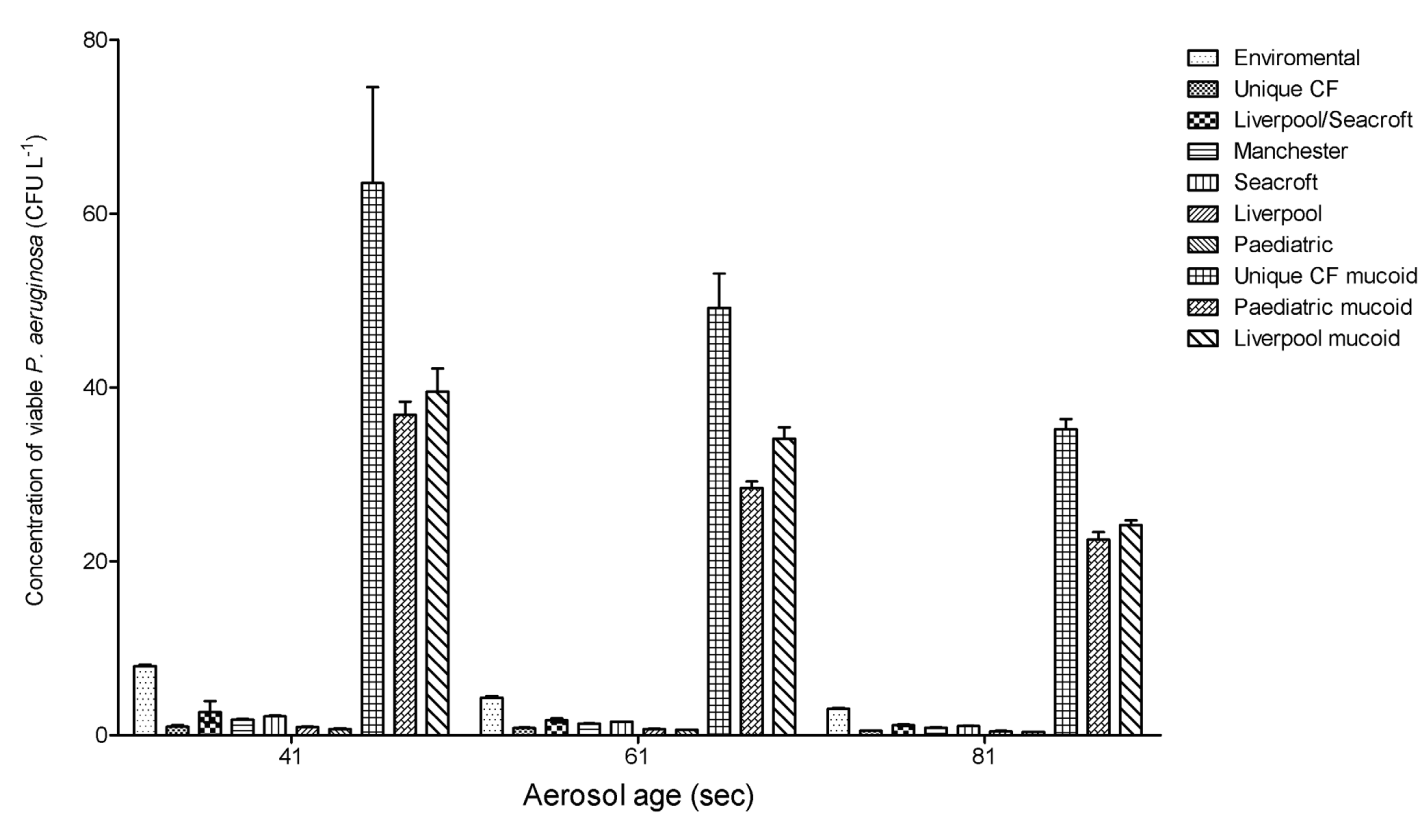
**Concentration of different strains of viable *P. aeruginosa *at different aerosol ages generated using Ringer's solutions.** Error bars represent standard error of mean.

The mucoid strains of *P. aeruginosa *produced aerosols containing significantly higher concentrations of viable bacteria than the non-mucoid strains (Liverpool p = 0.0181; Paediatric p = 0.0196; Unique CF p = 0.0264) (See Figure 7).

## Discussion

The laminar flow apparatus used in this study enabled comparison of recovered concentrations of viable bacteria, during steady state nebulisation, under controlled environmental conditions. In addition to concentration data, the study produced data regarding the size distribution of the aerosol particles containing viable bacteria in the apparatus. Liquid particles suspended in an aerosol are categorized as being either droplets or droplet nuclei. Droplets are generally considered to be greater than 10 μm in diameter [21], whereas droplet nuclei are airborne particles from which most of the liquid has evaporated and therefore less than 10 μm in diameter [22,23]. Droplets tend to fall to the ground quickly, whereas droplet nuclei have a low terminal velocity and therefore can potentially remain airborne for a number of hours. It has been postulated that Gram negative bacteria such as *P. aeruginosa *can only survive in droplets and not in droplet nuclei, the implication being that respiratory droplet nuclei are not implicated in the transmission of this pathogen [24]. The results of the laminar flow experiments suggest that this is not the case, as they demonstrate that *P. aeruginosa *can survive within droplet nuclei in artificially generated aerosols, with mucoid strains in particular surviving well in an aerosolised state. In so doing, we have demonstrated that aerial dissemination may be a plausible route of transmission for *P. aeruginosa *infection.

The Andersen 6-stage sampler is a standard piece of equipment and is probably the most widely used aerobiological sampler [25]. While our experiments demonstrated that it is possible to recover *P. aeruginosa *from bioaerosols using an Andersen 6-stage impactor, it is important to remember that the concentration cultured using the sampler may not be a true representation of the actual concentration of bacteria within the air. It has been estimated that the quantity of bacteria cultured from an air sample represents only approximately 10% of the actual bacterial burden, with the remaining 90% in a viable but non-culturable state [26]. Gram-negative bacteria are particularly vulnerable to the shear forces imposed during the sampling process. Consequently, it is generally thought that air samples in the clinical setting tend to underestimate the numbers of Gram-negative bacteria present in the air. Notwithstanding this, it is not known the extent to which these viable but non-culturable bacteria represent a potential risk for infection.

Our experiments demonstrated that the solution used for nebulisation is critical for the survival of the bacteria within aerosols. The use of hypotonic solutions produced a significant reduction in the ability of the bacteria to survive within the aerosol. Inoculation of bacteria into a hypotonic solution will result in an osmotic shock. However, in our experiments we found that this was not sufficient to kill the bacteria, as the control counts from the nebuliser remained stable pre- and post-nebulisation; it may however be that the osmotic shock is enough to leave the bacteria in a weakened state so that subsequent survival in an aerosol is significantly reduced. The reduction in survival may also have been due to the increase in the boiling temperature that occurs with the addition of a solute to a solution. An increase in the boiling temperature will result in a reduced rate of evaporation and hence protect the bacteria from the lethal effects of desiccation. The solution which allowed for the greatest survival of bacteria was 10% FBS. This solution will not only protect the bacteria from osmotic shock and desiccation, but also contains nutrients that will allow the bacteria to continue metabolising whilst suspended in the aerosol. In many respects this represents the case for bioaerosols produced by patients with CF, who will tend to liberate microorganisms in aerosols containing salts and proteinaceous material.

The environmental conditions of the aerosol are important for survival of bacteria within droplet nuclei. Bacteria are more rapidly desiccated in conditions of high temperature or low relative humidity. The data presented here confirms that the survival of *P. aeruginosa *is adversely effected by a small increase in temperature. However, increased humidity did not statistically improve the survival of *P. aeruginosa *within the aerosol. Both of these effects may have potential clinical implications as it would suggest patients with CF should avoid cool and damp air conditions in an effort to minimise infection with *P. aeruginosa*. Consideration should be made to the environmental air conditions when CF units are been designed. Potentially the use of warm and dry air may reduce the risk of cross-infection by *P. aeruginosa *between patients with CF. It would also seem reasonable to advise patients with CF to avoid the use of devices such as humidifiers as they may promote the survival of *P. aeruginosa *within aerosols.

The presence of a mucoid phenotype seems to be particularly important for the survival of *P. aeruginosa *with an aerosol. Isolates of the Liverpool, Leeds Paediatric and Unique CF strains expressing both a mucoid and non-mucoid phenotype were studied in the laminar flow apparatus. For all the strains the presence of a mucoid phenotype resulted in significantly increased concentrations in the aerosols. The mucoid strains over-produce the exopolysaccaride alginate which has been implicated in the persistence and pathogenesis of chronic *P. aeruginosa *infection in patients with CF [27]. The alginate may also improve the survival of the *P. aeruginosa *in aerosols by providing a protective barrier which reduces the lethal effects of desiccation upon the bacteria.

The data presented from this study provides support for the hypothesis that airborne dissemination may be important for the cross-infection of *P. aeruginosa *between patients with CF. There have been two studies that have demonstrated that patients with CF can produce aerosols containing viable *P. aeruginosa *[16,17] as well as a study demonstrating that *Burkholderia cepacia *complex can be disseminated from patients with CF during physiotherapy [28]. A further study only published in abstract form examining the frequency of bacterial shedding in the out-patients setting demonstrated that respiratory pathogens were more frequently cultured from the hands and airborne droplets of patients with CF than from environmental surfaces or equipment [29]. The sampling at the Liverpool CF unit demonstrated that the outbreak strain *P. aeruginosa *could be cultured from the air 3 hours after a patient had left the area. To survive this length of time suspended within an aerosol the bacteria must be in a droplet nuclei, because larger droplets would have fallen to the floor due the effect of gravity.

Although this study is limited in that the aerosol residency time for the laminar flow apparatus was relatively short at only just over 1 minute, this was however long enough for the bacteria to travel 4 m – conclusively demonstrating that the bacteria were transported in droplet nuclei and not in droplet form. The concentrations of *P. aeruginosa *used in the present study were lower than those commonly found in sputum samples from patients with CF which can contain approximately 10^7 ^CFU g^-1 ^[30]. Although it is theoretically possible that the inhalation of just a few bacteria might result in colonisation, the dose of *P. aeruginosa *required to cause pulmonary infection in patients with CF is not known

## Conclusion

There appears to be little difference in airborne survival between the epidemic and non-epidemic strains of *P. aeruginosa *expressing a non-mucoid phenotype. However the expression of the mucoid phenotype does seem important for survival within droplet nuclei. This suggests that particular emphasis should be made on segregating individuals free from *P. aeruginosa *from those with chronic infection who are more likely to be infected with mucoid strains of *P. aeruginosa*.

Finally, our study suggests that environment appears to influence bacterial survival and optimal conditions for reducing airborne transmission of *P. aeruginosa *between patients could include warming and drying the air within clinical areas and avoidance of humidification devices.

## Abbreviations

CF: Cystic fibrosis; CFTR: Cystic fibrosis transmembrane conductance regulator; FBS: Foetal bovine serum; NCIMB: National Collection of Industrial and Marine Bacteria; RH: Relative humidity.

## Authors' contributions

IJC undertook the studies, performed the statistical analysis and drafted the manuscript. LAF participated in the design of the study and assisted in undertaking the studies, CBB, MD, and DGP participated in the design and coordination of the study. All authors read and approved the manuscript.
